# Changes and Compromises in Health Choices during COVID-19 Lockdown in Kathmandu Valley: A Descriptive Cross-sectional Study

**DOI:** 10.31729/jnma.5790

**Published:** 2020-12-31

**Authors:** Carmina Shrestha, Sajan Acharya, Raksha Sharma, Roja Khanal, Jasmin Joshi, Calvin Ghimire, Prakriti Bhandari, Aditi Agrawal

**Affiliations:** 1 Patan Academy of Health Sciences-School of Medicine, Lalitpur, Nepal; 2 New York Medical College/Metropolitan Hospital Center, New York, USA; 3 Tesla Diagnostic Clinic, Kathmandu, Nepal; 4 Annapurna Neurological Institute and Allied Sciences, Kathmandu, Nepal

**Keywords:** *health choices*, *COVID-19*, *Nepal*, *pandemic*

## Abstract

**Introduction:**

Nepal government enforced a lockdown as a social distancing measure to curb the COVID-19 pandemic. The lockdown has led to compromises in day to day choices like food, exercise, sleep, self-care routines and utilization of healthcare facilities - directly and indirectly influencing aspects of health. Identification of compromised health choices can assist in better planning of inevitable future crises.

**Methods:**

This is a descriptive cross-sectional study based on an online self-administered questionnaire, done using CHERRIES criteria, conducted from March 30, 2020, to July 31, 2020 conducted in Kathmandu valley via convenience sampling. Ethical approval for the study was obtained from the Ethical Review Board of Nepal Health Research Council (Registration number: 2119; 300/2020 P). Descriptive statistics was used for analysis.

**Results:**

Our study had 340 (51%) female and 325 (48.7%) male participants. A total of 112 (67.9%) reported decreased consumption of tobacco and 178 (53.6%) reported decreased consumption of alcohol during the lockdown period. Participants who reported that they would have visited a hospital if they had a flu-like illness increased from 151 (22.6%) pre-pandemic to 391 (58.6%) post-pandemic. Increase in news consumption was reported by 528 (79.2%). Out of 43 (6.4%) participants with a chronic condition, 30 (69.8%) reported having missed follow up due to the lockdown.

**Conclusions:**

The health of an individual is determined by various choices s/he makes on a day to day basis. Many of those choices are in turn influenced by the availability and accessibility of commodities. Lessons learned from the affected lives due to COVID-19 can be used in proper planning of inevitable future crises.

## INTRODUCTION

In Nepal, the government decided to go on lockdown as a non-pharmacological intervention which began on 24^th^ March 2020 and was extended up to 21^st^ July 2020.^1^

The lockdown led to various lifestyle modifications and a direct and indirect influence on the health of individuals. Isolation measures enforced to curb the pandemic have affected both the physical and mental health of individuals.^2–4^ Demographic characteristics like age,^5^ gender,^4^ education,^6^ household size, age of the most elderly member of the family can all influence how a family perceives the effect of COVID-19 on various aspects of health. The lockdown has led to compromises in day to day choices like food,^7^ exercise, sleep^8^ and self-care routines.

This study was carried out to identify the changes and compromises in health choices due to the lockdown in Kathmandu valley. Identification of compromised health choices can assist in planning inevitable future crises.

## METHODS

This is a descriptive cross-sectional study based on an online self-administered questionnaire conducted from March 30, 2020 to July 31, 2020 in Kathmandu valley. Ethical approval for the study was obtained from the Ethical Review Board of Nepal Health Research Council, Kathmandu, Nepal (Registration number: 2119; 300/2020 P).

The sample size was calculated using the formula given below:

$$\begin{array}{ll} \text{n} & \text{= }{\text{Z}}^{\text{2}}\times \text{p}\times \text{(1}-\text{p)}/{\text{e}}^{\text{2}}\\ & \text{= }{\left(1.96\right)}^{\text{2}}\times 0.50\times 0.50/{(\text{0.08)}}^{\text{2}}\\ & \text{= }600 \end{array}$$

Where,

n = required sample size,
Z = 1.96 for Confidence Interval at 95%,
p = Prevalence, 50% (for maximum sample size)
e = margin of error as 8%

Taking a non-response rate of 10%, the sample size becomes 660.

$$\begin{array}{ll} \text{N} & \text{= 600}+10\%\text{ of }600\;\\ & \text{= }600+60\\ & \text{= }660 \end{array}$$

Hence, we took 667 participants in our study using convenient sampling technique.

Due to its web-based nature we used the CHERRIES checklist to improve the quality of the study.^9^ The study population consisted of all the individuals currently living in lockdown in Nepal. The self-administered questionnaire was created in google form. The questions regarding health choices were made by a team of medical doctors and reviewed by a psychiatrist. Translation of the questionnaire to Nepali language was done by the investigators themselves and reviewed by a translator holding a Masters of Art degree in Nepali. Pre-validation was done by administering both English and Nepali questionnaires using a form that stops receiving input after 20 completions. Convenient sampling was used excluding research team members and pre-test participants.

The data collection was done online by sending self-administered questionnaires via email to anyone willing to participate in the study. The questionnaire was also advertised in different social media platforms clearly stating the purpose of study and no benefits from participation. Informed consent from willing participants was obtained through the same google form. Participants could review and change their answers before submitting. A request to close the questionnaire if the participant thinks she has already filled the form was mentioned before informed consent. The length of the study period was 4 months (March 30, 2020, to July 31, 2020). The data was analysed using frequency and percentages in SPSS version 23.0. A total of 669 questionnaires were received; however, 2 incomplete questionnaires were excluded from the analysis.

## RESULTS

Out of the total 667 participants, 340 (51%) were female, 325 (48.7%) were male. Half the participants (52.9%) were between 18 to 25 years of age. The majority of the participants were single (73.3%). More than half of the participants (62.8%) were educated up to the bachelor's level. Four hundred and two (60.3%) participants were health workers. The median number of people living together during the lockdown was 4 with an IQR of 2. A total of 228 (34.1%) participants were living with an elder member aged 60 and above. Details of demographics are provided in Table 1.

**Table 1:** Socio-demographic characteristics of study participants (N=667).

Categories	n (%)
Age (in years)	
18 to 25	353 (52.9)
26 to 35	231 (34.6)
36 to 45	30 (4.5)
46 to 55	33 (4.9)
56 to 65	17 (2.5)
66+	3 (0.4)
Sex^*^	
Male	325 (48.7)
Female	340 (51.0)
Marital status^†^	
Single	489 (73.3)
Currently Married	170 (25.5)
Education	
Schooling not completed	3 (0.4)
SLC, 10+2 or equivalent	123 (18.4)
Bachelor’s degree	419 (62.8)
Master’s degree or above	122 (18.3)
Household size	
1	20 (3.0)
2	54 (8.1)
3	114 (17.1)
4	204 (30.6)
5	110 (16.5)
≥ 6	165 (24.7)
Age of eldest family member	
< 50	237 (35.5)
50 to 59	202 (30.3)
60 to 69	104 (15.6)
70 to 79	73 (10.9)
80+	51 (7.6)

* Two preferred not to report their gender.

† Five participants were widowed and three were divorced.

**Use of helpline and sources of information related to COVID-19**

One-third of the participants i.e., 230 (34.5%) relied on social media to get information about COVID-19. Other sources of information included World Health Organization i.e., 151 (22.6%), radio/television i.e., 99 (14.8%), Ministry of Health and Population Nepal i.e., 90 (13.5%), online news portals i.e., 83 (12.4%) and others i.e., 14 (2.1%). More than half of the respondents i.e., 398 (59.7%) reported that they had noted down telephone numbers of helplines on COVID-19 for use during emergencies. However, only 136 (20.4%) reported utilizing the helpline. The majority of the participants i.e., 528 (79.2%) said that their news consumption increased during lockdown followed by 104 (15.6%) who reported no change and 35 (5.2%) who reported a decrease.

**Utilization of health facilities during the lockdown**

Out of 43 (6.4%) participants with a chronic condition, 30 (69.8%) reported having missed follow up due to the lockdown. When questioned regarding healthcare institute visits during lockdown compared to before, only 60 (9%) participants reported increased visits while 244 (36.6%) claimed to have decreased hospital visits and majority 363 (54.4%) reported no change.

**Participant perception regarding flu symptoms**

Out of 667 participants, 235 (35.2%) claimed that they used to ignore flu symptoms before the COVID-19 pandemic. Three hundred and fifty (52.5%) participants reported increased fear regarding flu-like symptoms during the lockdown while 264 (39.6%) reported no change and 53 (7.9%) reported decreased fear about flu symptoms. Before the pandemic, 151 (22.6%) of the participants reported visiting health care centers when they developed flu-like symptoms. However, following the pandemic, 391(58.6%) participants claimed that they would visit the hospital if they developed any flu-like symptoms.

**Social Interaction during COVID-19**

Majority of the participants (69%) reported an increased feeling of connectedness with family members and friends during lockdown, 167 (25%) said that there was no change and 40 (6%) reported a decrease. Most of the participants (50.5%) reported no change in maintenance of privacy during lockdown followed by 173 (25.9%) participants who reported decreased privacy and 157 (23.5%) participants who reported an increased maintenance of privacy. A total of 351 (52.6%) participants were worried about not being able to visit relatives during COVID-19.

**Personal care practices during COVID-19**

Majority of the participants reported an increase in personal hygiene (82.3%), exercise routine (54.1%), selfcare routine (56.1%) and involvement in creative projects (67.8%) during the lockdown. Most of the participants reported an increase in their weight (45.3%). Over 579 (86%) of the participants claimed to have increased their use of technologies (mobile, TV, laptops) during the lockdown.

**Table 2:** Change in personal care practices during lockdown (N=667).

Variables	Decreased	Increased	Not Changed
	n (%)	n (%)	n (%)
Personal hygiene	12 (1.8)	549 (82.3)	106 (15.9)
Exercise	103 (15.4)	361 (54.1)	203 (30.4)
Selfcare routine	67 (10.0)	374 (56.1)	226 (33.9)
Creative projects	49 (7.3)	452 (67.8)	166 (24.9)
Quality of diet	61 (9.1)	451 (67.6)	155 (23.2)
Sleep duration	73 (10.9)	420 (63.0)	174 (26.1)
Weight	73 (10.9)	302 (45.3)	292 (43.8)
Use of Mobile/TV/Laptop	23 (3.4)	579 (86.8)	65 (9.7)

**Substance use during lockdown**

Among participants who had never smoked or drank alcohol, 19 (5.7%) participants started drinking and 5 (1%) of the participants started smoking during the lockdown respectively. Among 165 (24.7%) participants who smoked occasionally or often, 112 (67.9%) reported decreased consumption of tobacco during the lockdown period. Similarly, out of 332 (49.8%) participants who consumed alcohol occasionally or often, 178 (53.6%) reported decreased use during lockdown. Details of substance use patterns are provided in Table 3.

**Table 3:** Change in substance use pattern during the lockdown period (N=667)

n (%)	Before Lockdown Increased n (%)	During Lockdown	
		Decreased n (%)	Not changed n (%)
Smoking				
Never	502 (75.3)	5(1.0)	-	497 (99.0)
Sometimes	102 (15.3)	8 (7.8)	69 (67.6)	25 (24.5)
Often	63 (9.4)	8 (12.7)	43 (68.3)	12 (19.0)
Drinking				
Never	335 (50.2)	19 (5.7)	-	316 (94.3)
Sometimes	306 (45.9)	21 (6.9)	168 (54.9)	117 (38.2)
Often	26 (3.9)	7 (26.9)	10 (38.5)	9 (34.6)

**Pattern of stockpiling during lockdown**

Over 474 (71%) of the participants were worried about food, essential goods and medical supply being unavailable during the pandemic. Majority of the participants kept the usual stock of medication (60.4%) and food/essential goods (34.9%). Stockpiling of food and essential supplies was more prevalent in participants who were worried about the unavailability of supplies during the pandemic. Change in pattern of keeping reserves of supplies is described in Table 4.

**Table 4:** Reported stockpiling of goods (as measured by time it would last) (N=667).

Stock of	Worried about availability	Stock lasting				
		As usual	2 weeks	1 month	3 month	> 6 month
		n (%)	n (%)	n (%)	n (%)	n (%)
Food and essential goods	Yes	155 (32.7)	117 (24.7)	163 (34.4)	33 (7.0)	6 (1.3)
	No	78 (40.4)	44 (22.8)	54 (28.0)	16 (8.3)	1 (0.5)
Medication	Yes	281 (59.3)	66 (13.9)	98 (20.7)	27 (5.7)	2 (0.4)
	No	122 (63.2)	28 (14.5)	33 (17.1)	8 (4.1)	2 (1.0)

## DISCUSSION

The coronavirus disease 2019 (COVID-19) was first reported in Wuhan, China on 31st December 2019 after a patient presented with an unknown case of pneumonia. WHO declared it as a public health emergency on 30th Jan 2020 and later declared it as a pandemic on 11th January 2020.^10^ Lifestyle, eating habits, social connections and day to day activities of Nepalese population changed during the COVID-19 lockdown. Some of these changes impact health directly or indirectly. For this study "health choices" meant choices an individual or a family make on a daily basis that influences their health directly or indirectly. Choosing what to eat, whether or not to exercise, the extent of consumption of news, the spectrum of social interactions can all fall under the umbrella of health choices. The perceived change in type of diet, availability and stockpiling of food and medication, exercise routines, personal hygiene, sleep duration, substance use, social interactions, and healthcare usages have been corroborated by many studies.^5,8,11,12^ Lockdown is a social distancing measure. In a study done by Medeiros et al to evaluate the effectiveness of strict social distancing measures applied in two Chinese provinces in reducing the incidence and mortality from COVID-19, strict social distancing measures represented an effective way to slow the progression of COVID-19 epidemics. However, these measures have a great economic, psychological and social impact.^13^

According to an Italian survey done to assess eating habits and lifestyle changes during COVID-19 lockdown, the perception of weight gain was observed in 48.6% of the population^12^ which is comparable to reported increased weight in our study (45.3%). Noteworthy is the fact that individuals with obesity are at increased risk of either chronic or acute diseases, which includes COVID-19 infection and complications.^14^ The same study also reported that in Italy 3.3% of smokers decided to quit smoking^12^ which contrasts our finding that 1% of the participants started smoking during lockdown. A decrease in the frequency of smoking (67.9%) and drinking (53.6%) was observed which may reflect decreased accessibility or behavioral change secondary to fear of COVID-19.

Our study demonstrated that over one third of our study participants (34.5%) utilized social media as a primary source to gain information related to COVID-19. False information and myths regarding the pandemic is likely to instill fear and anxiety among the viewers.^15^ However, these social media platforms can be instead utilized by authentic sources to maximize spread of reliable information as well as limit and falsify misinformation and rumors. Additionally, 71% of our study participants were worried about food, essential supplies and medications being unavailable during lockdown. Even before the pandemic, food insecurity and malnutrition was prevalent in Nepal. The stay at home orders to combat COVID-19 is likely to further aggravate the situation.^16^ Appropriate measures need to be taken to ensure adequate distribution of food and essential supplies during lockdown especially to those who live on daily wages and are most affected by isolation orders. Furthermore, steps should also be taken to curb the spread of misinformation regarding food shortages which automatically leads to hoarding of supplies by those who can afford, instead creating temporary shortages. The Federal Emergency Management Agency (FEMA),^17^ recommends maintaining a two-week supply of food, water and essentials during emergencies; whereas Save the Children,^18^ suggests that households consider stockpiling a 6-week supply of essential items. Country specific guidelines regarding storage of essentials during this pandemic should be issued.

Fever, cough, and fatigue are the most common symptoms presented by COVID-19 patients.^19^ This symptom overlap with flu can confuse the patients and increase their anxiety. That can be one of the reasons why the number of participants who would visit the participants if they had a flu-like illness increased from 151 (22.6%) pre-pandemic to 391 (58.6%) post-pandemic. Along with the lockdown implemented by the government all the health services providing modalities have been changed. The Out-Patient Department (OPD) has been closed and non-emergency services have been postponed. People were also advised to seek medical care only in the case of emergency. As many as 69.8% of people with pre-existing chronic conditions could not go for their regular follow up during the lockdown. A substantial investment had been made in the health sector to achieve the current rate of utilization. A longitudinal follow up study on if or not the poor utilization of health services for chronic health problems forced by the current pandemic persist can shed light to the long term effect.

A reflective article from Canada after the SARS epidemic of 2003 concluded that quarantine seriously disrupts lives, isolates individuals from the outside world, and jeopardizes workers' livelihood unless appropriate compensation is available.^20^ An understanding of what health choices are perceived to be compromised can help formulate policies and directives regarding information dissipation and the provision of compensation. Only 34 (5.1%) participants disagreed that the lockdown would positively impact the society in the future. This encourages the hope that the deserved attention will duly be provided to the preparedness aspects of inevitable future crises. However, as the COVID-19 pandemic is ongoing, our data needs to be confirmed and investigated in future with more extensive population studies. Also, the majority of our study participants were between 18 to 25 years of age; hence, the perceived compromised health choices may represent that of a younger population. Additional studies incorporating a wider age group is required.

## CONCLUSIONS

Health of an individual is determined by various choices s/he makes on a day to day basis. Many of those choices are in turn influenced by availability and accessibility of commodities. Lockdown, used as a measure for enforcing social distancing can thus change health choices that people make. Patterns like decrease in smoking or drinking, probably due to difficult accessibility, can be leveraged to design policies that can impact public health positively. Lessons learnt from the affected lives due to COVID-19 can be used in proper planning of inevitable future crises.
